# The 'nightmare' of wrong level in spine surgery: a critical appraisal

**DOI:** 10.1186/1754-9493-6-14

**Published:** 2012-06-19

**Authors:** Claudio Irace, Susanna Usai

**Affiliations:** 1 Dept of Neurosurgery, Hospital IGEA, Via Marcona 69, Milan, 20129, Italy; 2 Headache Center, Neurological Institute “C. Besta” IRCCS Foundation, Milan, Italy

**Keywords:** Discectomy, Incorrect site surgery, Interspinous device, IRACE method, Wrong level, Wrong level spine surgery

## Abstract

The recent article published in the Journal by Lindley and colleagues (Patient Saf. Surg. 2011, 5:33) reported the successful surgical treatment of a persistent thoracic pain following a T7-8 microdiscectomy, truly performed at the ‘level immediately above’. The wrong level in spine surgery is a multi-factorial matter and several strategies have been designed and adopted to try decreasing its occurrence. We think that three of these factors are crucial: global strategy, attention, precision in level identification; and the actors we identified are the surgeon, the assistant nurse and the (neuro)radiologist respectively. Basing upon our experience, the role of the radiologist pre- and intraoperatively and the importance of the assistant nurse are briefly described.

## Background

In their recent article Lindley and Colleagues reported the successful surgical treatment of a persistent thoracic pain following a T7-8 microdiscectomy, truly performed at the ‘level immediately above’ [1].

First of all we greatly appreciated the lucid and meticulous management of such a case, which is peculiar because of its unusual anatomy and the history of a previous surgery at the same level; although it’s beyond the scope of this letter, probably we’d have opted for a repeat standard microdiscectomy at the correct level without attempting a posterior fusion, even though a concomitant rib fracture had been diagnosed preoperatively. Then, the attention paid to those medico-legal implications, which are deeply and insidiously linked to this case, is well deduced: the elucidation of the rarity of the thoracic spine anatomy of this patient is reported in a so detailed manner, that this case report is not only scientifically interesting, but it may also result as a powerful defence of those surgeons who performed the previous discectomy. Basing upon our experience in the matter of wrong level in spine surgery we wish to add some observations.

## Team working: the spine surgeon and the (neuro)radiologist

Lindley and coauthors ‘recommend working closely with radiology colleagues’ and support ‘weekly radiology conferences…to assist with preoperative planning’ [1]. We do agree with them concerning this team work: in addition, we consider the presence of the (neuro)radiologist in the operating theatre, or the remote interpretation of neuroimaging he can provide, really helpful for different reasons. Patient’s local conditions such as scoliosis, obesity, coexistence of internal metallic instrumentation easily obscure the radiographic appearance; the radiologist is the one who can correctly extricate among all different ‘greys’. Moreover the radiologist is free from surgery’s stressing pressure, as on the contrary the surgeon is: therefore he can provide a correct, illuminating and ultimate interpretation of intraoperative radiographic images (or ask for repeating it, if required) stressless. Lindley and Colleagues raise another crucial point of interest: the adoption of a common system to identify spinal pathological levels [1]. A common system for level identification would be ideal in theory, but its practical application is rather problematic; our experience, as Italian regional and extra-regional referral center for spine diseases, showed us that patients coming to our attention, have received multiple neuroimaging reports, often conflicting one another. This condition is further blurred by the utilization of different, often confounding, terms to describe anatomical variations: ‘vestigial’, ‘rudimental’, ‘partial lumbarization or sacralization’, ‘last useful disc space’, variously mixed; to mitigate further confusion our strategy, both at the moment of compilation of medical charts and when describing surgical report, is to call the pathological (operated!) spine level exactly as the main neuroimaging report really does.

## The nightmare of the wrong-level: how to avoid it

Concerning protocols adopted to avoid a wrong-level spinal operation, in addition to the ‘Sign, Mark and Xray (or SMaX) Program’ [2] and the ‘JCAHO Protocol’ [3] reported by the Authors, we wish to mention two more strategies (although not ‘national’, they received recent wide scientific attention): the ‘ABCD pause’ [4] and the IRACE (Intraoperative Radiograph And Confirming Exclamation) method [5]. If we compare all these strategies, we can observe that concerning the ‘SMaX Program’, our method seems more detailed and integrated because of the subsequent oral confirmation. The ‘JCAHO Protocol’ appears effective when applied to other fields of surgery, but it is less specific than the ‘IRACE method’ when used in lumbar spine surgery. The ‘ABCD pause’ does not identify the single person dedicated to the oral check; moreover the time-out does not address the problem of level error which may derive from incorrect direction of dissection during microsurgery. Although the attention paid by Lindley and Coll. was directed obviously to avoidance of wrong site surgery at the thoracic region, which indeed may be considered a pure surgical problem, other factors identified as crucial to decrease the rate of wrong level spinal (usually lumbar) operations must be remembered once again. Fatigue, sense of inferiority, external forces pressing the whole surgical team to complete a crowded operative session quickly, or a mixture of them, represent an explosive cocktail; and this is even more true, if you consider the current occurrence of repeat lumbar spine operations, strongly resembling one another, scheduled on the same day: such a scenario is not so dissimilar from an assembly line, which implies a high potential for gross error [6].

**Figure 1:**
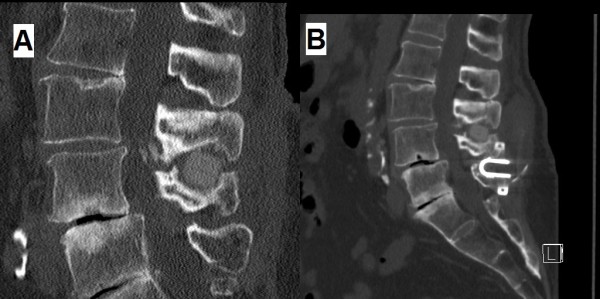
**Case example of wrong level spine surgery. Sagittal reformatted CT scan of the lumbar spine; this patient came to our attention after having performed an ‘interspinous stabilization L4-5’ elsewhere, as reported in medical discharging chart; soon after surgery her bilateral *claudicatio radicolaris *started to worsen. **A. the preoperative study clearly shows the DIAM^®^ interspinous device applied at the L3-4 interspinous space (wrong). B. in the postoperative image the COFLEX^®^ device correctly inserted at L4-5 is visualized; the DIAM^®^ at L3-4 was intentionally left in place to avoid late compromise of segmental stability.

Of course the analysis of those factors deemed crucial in the occurrence of exploration of a wrong level in spinal surgery must be included in a wider evaluation of the context in which wrong site procedures occur. Recently a retrospective analysis of a prospective database covering a 6½-year period found 5 cases of wrong level spinal surgery in a total of 27370 physician self-reported adverse occurrences; these 5 out 27370 cases (~ 0.02%) had a ‘significant harm’ [7]. Although this rate of error may appear very low, it must be pointed out that perhaps other similar cases could have had ‘minimal harm’ or passed away unmentioned. In addition, incidentally this study sheds another interesting light on the role of the radiologist; if the total rate of wrong site events is considered, the disciplines of orthopaedic surgery and anaesthesiology are the most involving (22.4 and 12.1%) along with general surgery (16.8%), as radiology is minimally reported (3.7% only) [7]. In our opinion these data, if confirmed, could encourage a more active role of (neuro)radiologists, who indeed make less errors, in the whole process dedicated to the avoidance of wrong site operations; Lindley’s final statement that a preoperative consultation with radiologists is precious to identify unconventional spinal segmentation, may be read as an additional support to this opinion too. In any case, concerning lumbar spine one-level operations, including endoscopic procedures (a Japanese multicenter study collected 6 cases of ‘wrong level surgeries’ in a group of 6239 spinal endoscopies, about 0.1%, performed on 2007 [8]) and interspinous stabilizations (Figure 1) too, we think that an intraoperative fluoroscopic confirmation by means of a wire placed in the cranial spinous process of the level to be operated on, is a fundamental step to try avoiding a wrong site surgery.

## Competing interest

The Senior Author (CI) declares that the “IRACE method” reported above represents his own method [5]; the Junior Author (SU) declares that she has no competing interests.

## Authors’ contribution

CI wrote the letter and SU contributed to its revision. All authors read and approved the final manuscript.
